# Distribution of Functional CD4 and CD8 T cell Subsets in Blood and Rectal Mucosal Tissues

**DOI:** 10.1038/s41598-019-43311-6

**Published:** 2019-05-06

**Authors:** Praveen Kumar Amancha, Cassie G. Ackerley, Chandni Duphare, Mark Lee, Yi-Juan Hu, Rama R. Amara, Colleen F. Kelley

**Affiliations:** 1 The Hope Clinic of the Emory Vaccine Research Center, Division of Infectious Diseases, Department of Medicine, Emory University School of Medicine, Decatur, GA 30030 United States; 2 Present Address: Pfizer Pharmaceuticals, Cambridge, MA United States; 3 Yerkes National Primate Research Center, Emory Vaccine Center, Atlanta, GA 30329 United States; 4 Department of Biostatistics and Bioinformatics, Rollins School of Public Health, Emory University, Atlanta, GA 30322 United States

**Keywords:** Mucosal immunology, HIV infections

## Abstract

A better understanding of the distribution and functional capacity of CD4 T helper (Th) and CD8 T cytotoxic (Tc) cell subsets in the rectal mucosa (RM), a major site for HIV acquisition and replication, in adults is needed. In this study, we compared the distribution of Th and Tc cell subsets between blood and RM compartments in 62 HIV negative men, focusing primarily on IL-17-producing CD4 and CD8 T cells due to their importance in establishing and maintaining mucosal defenses, and examined associations between the frequencies of Th17 and Tc17 cell subsets and the availability of highly HIV-susceptible target cells in the RM. The RM exhibited a distinct immune cell composition comprised of higher frequencies of Th2, Th17, and Tc17 cells compared to the peripheral blood. The majority of Tc17 cells in RM were quadruple-cytokine producers (IL-17A^+^, IFN-γ^+^, TNF-α^+^, and IL4^+^), whereas most Th17 cells in blood and RM were single IL-17A producers or dual-cytokine producers (IL-17A^+^TNF-α^+^). In a separate cohort of 21 HIV positive men, we observed similar tissue distributions of Th and Tc cell subsets, although Tc17 cell frequencies in both blood and tissues were very low. Higher frequencies of multi-cytokine-producing Th17 and Tc17 cells in RM of HIV negative men positively correlated with increased mucosal HIV target cells, suggesting a need to further characterize the effector functions of these cells and their role in HIV acquisition and pathogenesis.

## Introduction

The lower gastrointestinal (GI) tract is an important site for HIV transmission, replication, and subsequent systemic viral dissemination following both direct inoculation through receptive anal intercourse as well as secondary spread following initial infection from distant mucosal or systemic sites^1^. Thus, the intestinal mucosal immune environment likely plays a critical role in HIV susceptibility and in the pathogenesis of chronic infection^2^. Unlike other mucosal sites, the lower GI tract is distinct in that it contains the largest lymphoid compartment of the immune system, accounting for more than 60% of all T cells in the human body^3^. Previous studies have demonstrated significant differences in the composition and tissue compartmentalization of naïve and memory CD4/T helper (Th) and CD8/T cytotoxic (Tc) cell subsets between peripheral blood, lymphoid tissues, and mucosal sites^4–6^. However, there is little known about potential differences in the distribution of Th and Tc subsets between the peripheral blood and rectal mucosal compartments based on their effector functions. Defining the distribution, phenotype, and functional profiles of CD4 and CD8 T cells within the rectal mucosa may provide a better understanding of immunologic factors that contribute to HIV transmission and pathogenesis in the rectal mucosa following host exposure.

Activation by an antigen-presenting cell in the presence of specific cytokines stimulates the differentiation of naïve CD4 T cells into a specific effector T helper subset, e.g. Th1, Th2, Th17 and Treg cells^7^. These subsets are defined by the expression of characteristic lineage-determining transcription factors and by the secretion of distinct cytokines that mediate their effector functions^8^. Th17 cells abundantly populate mucosal sites where they contribute to protective immunity, while also maintaining the integrity of the mucosal epithelial barrier. These cells are integral for the clearance of extracellular bacterial and fungal pathogens through secretion of two primary isoforms of IL-17, IL-17A and IL-17F. These cytokines serve to recruit and activate neutrophils in response to pathogenic invasion at mucosal barrier surfaces^9^. In contrast to this pro-inflammatory role, Th17 cells may also produce IL-22, a cytokine that promotes preservation and restoration of the gut epithelial tight junctions thereby reducing gut permeability^10^. During acute HIV infection, a massive depletion of CD4 T cells occurs in mucosal tissues with Th17 cells being preferentially targeted and infected. The loss of Th17 cells subsequently increases the vulnerability of the mucosal barrier to injury, leading to gut microbial translocation, which is thought to promote a chronic immune activation state in HIV-infected individuals^11–13^.

In a healthy host, a steady state of controlled inflammation exists in the intestinal mucosa allowing for a balance between protective immunity against pathogens and tolerance to self-antigen and commensal bacteria^14^. Aberrant immune responses can result in the development of pathological states of excess inflammation. The overexpression of pro-inflammatory cytokines, such as TNF-α and IFN-γ, by Th17 cells has been implicated in the development of several autoimmune and inflammatory diseases, including rheumatoid arthritis, multiple sclerosis, and inflammatory bowel disease^14–16^. Unlike Th1 and Th2 subsets, the differentiation of Th17 cells demonstrates lineage plasticity, or an ability to acquire new effector functions associated with other T cell lineages^11^. For instance, exposure of Th17 cells to IL-12 will induce the co-expression of transcription factors that promote the differentiation of Th1 and Th17 cells, leading to the production of Th17 cells with Th1 features. These Th17/Th1 cells are considered pro-inflammatory and pathogenic as they were first discovered in the context of experimental autoimmune encephalomyelitis murine models and have been shown to contribute to autoimmune disease pathogenesis^11^. The role of these Th17/Th1 cells in HIV acquisition is currently under investigation with some *in-vitro* studies demonstrating an increased susceptibility to HIV infection^12,17^.

CD8 T cells are similarly diverse in their capacity to differentiate into distinct functional phenotypes. The cytokines produced by the Tc subsets, i.e. Tc1, Tc2, and Tc17 cells, mirror those secreted by their CD4 counterparts. HIV transmission triggers the activation and differentiation of CD8 T cells, which results in a robust cytotoxic response, primarily from Tc1 cells, that fails to prevent infection but does serve to slow disease progression^18^. Tc17 cells, a more recently discovered and less well characterized CD8 T cell subset, share important features with Th17 cells. Both subsets predominate in the intestinal mucosa, secrete IL-17A, and appear to play a role in protecting intestinal mucosal integrity. In prior studies, Tc17 cells have demonstrated the capacity to produce multiple cytokines, including IL-2 and TNF-α, while exhibiting few cytotoxic effects, compared to Tc1 and Tc2 cells, as they lack expression of perforin and granzyme B^19–21^. At this time, there is a paucity of information about the effector functions of these cells and their role in host defense against viral pathogens, including HIV.

In this study, we sought to determine the frequency, phenotype, and functional profiles of CD4 and CD8 T cell subsets in the peripheral blood and rectal mucosal tissue compartments of healthy HIV negative men, focusing primarily on the IL-17A-producers, Th17 and Tc17 cells. In addition, we examined the tissue distribution of these cell subsets in a separate cohort of HIV positive men with preserved peripheral blood CD4 counts. We hypothesized that the composition and functional activity of CD4 and CD8 T cell subsets would be distinct within the blood and rectal mucosal tissue compartments.

## Results

### Th2, Th17 and Tc17 cell subsets are predominant in the rectal mucosa compared to peripheral blood of HIV negative men

**Figure 1:**
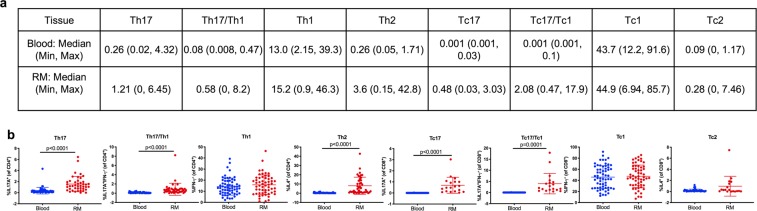
CD4 (Th) and CD8 (Tc) T cell cytokine-producing subsets in blood and rectal mucosa (RM) of HIV negative men. Definition of subsets: CD4 + IL-17A + were identified as Th17, CD4^+^IFN-γ^+^ as Th1, and CD4^+^IL-4^+^ as Th2 cells. IL-17A and IFN-γ-producing T cells were identified as Th17/Th1 cells. The same definitions were utilized for the CD8 T cells based on cytokine production. (**a**) Median, minimum and maximum frequencies of CD4 and CD8 T cell subsets expressed as percentages. (**b**) Scatter plots of the defined CD4 and CD8 T cell subsets in blood (blue circles) and RM (red circles). Error bars represent means ± SD; statistically significant findings were considered when p < 0.05 (Wilcoxon matched-pairs signed rank test).

We investigated the frequencies of IFN-γ-, IL-4-, and IL-17A-producing CD4 and CD8 T cells in blood and rectal mucosa from 62 healthy, HIV negative men to determine if there are compartmental differences in the distribution of these cell subsets. Isolated mononuclear cells from peripheral blood and rectal mucosal samples were stimulated with phorbol myristate acetate (PMA) and Ionomycin to induce cytokine production by the total T cell population. Using an intracellular cytokine assay and multi-color flow cytometry, the levels of IFN-γ-producers (Th1 or Tc1 for IFN-γ-secreting CD4 or CD8 T cells, respectively), IL-4-producers (Th2 or Tc2 for IL-4-secreting CD4 or CD8 T cells, respectively), and IL-17A-producers (Th17 or Tc17 for IL-17A-secreting CD4 or CD8 T cells, respectively) were quantified (Fig. S1). The median frequencies of Th17 (1.21 vs 0.26; p < 0.0001), Th2 (3.6 vs 0.26; p < 0.0001), and Tc17 (0.48 vs. 0.01; p < 0.0001) subsets were significantly higher in the rectal mucosa compared to the blood compartment (Fig. 1). There were overall very few IL-4- (Tc2) and IL-17A- (Tc17) producing CD8 T cells observed in the peripheral blood. The frequencies of Th1 and Tc1 cells were comparable between the peripheral blood and rectal mucosa. In addition, the frequencies of pro-inflammatory Th17/Th1 and Tc17/Tc1 cells producing both IL-17 and IFN-γ were significantly higher in the rectal mucosa compared to the blood (p ≤ 0.0001 for both comparisons).

**Figure 2:**
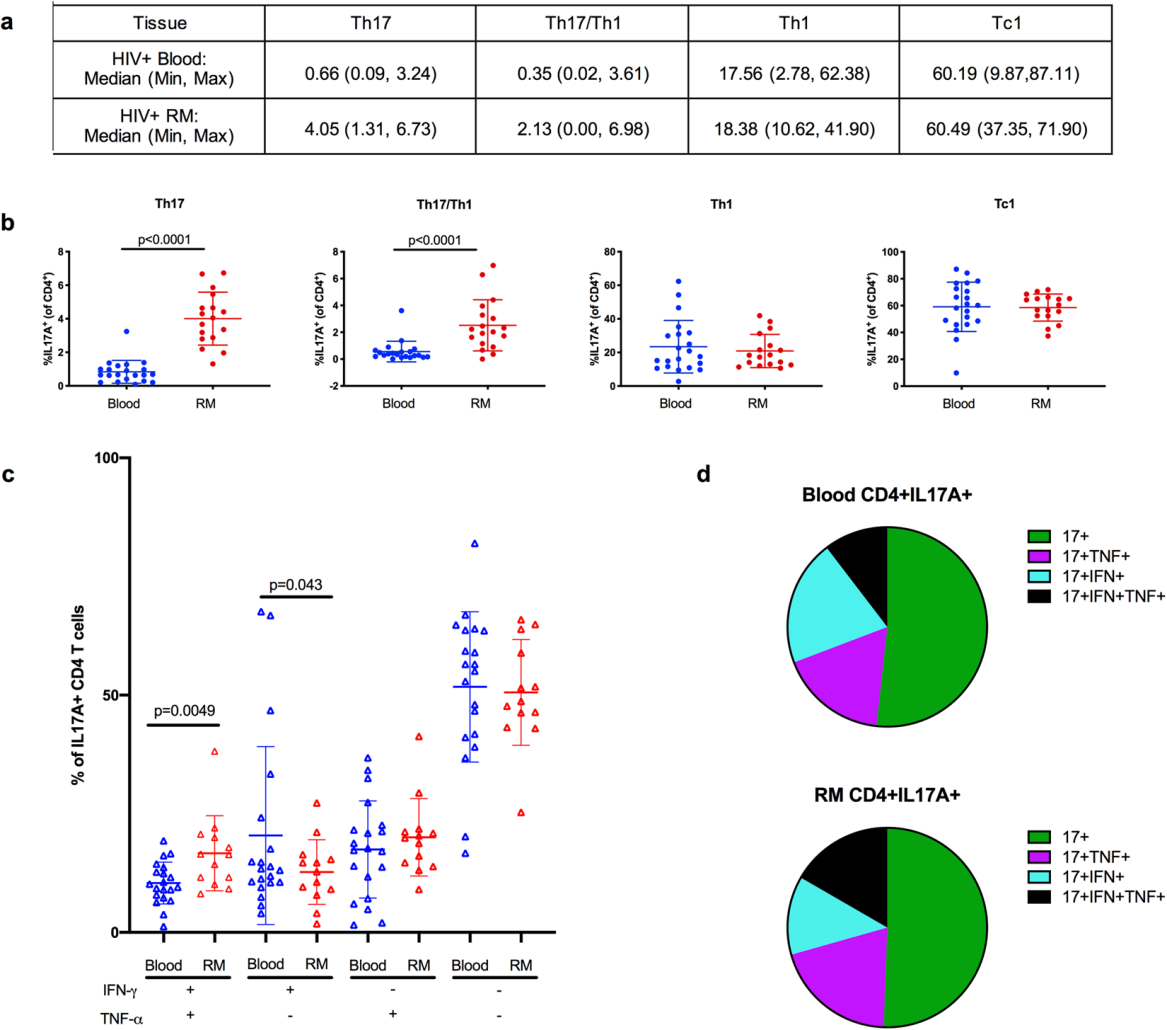
The distribution of CD4 and CD8 T cell cytokine-producing subsets and polyfunctionality of IL-17-producing CD4 T cells in blood and rectal mucosa (RM) among HIV positive men. Definition of subsets: CD4 + IL17A + were identified as Th17, CD4^+^IFN-γ^+^ as Th1, and CD8^+^IFN-γ^+^ as Tc1 cells. IL-17A and IFN-γ-producing T cells were identified as Th17/Th1 cells. (**a**) Median, minimum and maximum frequencies of CD4 and CD8 T cell subsets expressed as percentages. (**b**) Scatter plots of the defined CD4 and CD8 T cell subsets in blood (blue circles) and RM (red circles). (**c**) Comparison of the frequencies of polyfunctional Th17 cells between blood (blue triangles) and RM (red triangles). Cytokine expression profiles are based on TNF-α and IFN-γ secretion by IL-17A-producing CD4 T cells. (**d**) Pie graphs representing data from (**c**) demonstrating that the majority of Th17 cells are single IL17A-producers in the blood and rectal mucosa. Error bars represent means ± SD; statistically significant findings were considered when p < 0.05 (Wilcoxon matched-pairs signed rank test).

In a separate analysis, we compared the distribution of single cytokine-producing Th1, Th17, and Tc1 cells and pro-inflammatory Th17/Th1 cells producing both IL-17 and IFN-γ between blood and rectal mucosal tissues in a cohort of 21 HIV positive men who have sex with men with preserved peripheral blood CD4 counts (CD4 > 350 cells/μl; 13/21 (62%) with HIV viral load <20 copies/ml on anti-retroviral therapy). Of note, these results cannot be directly compared to those observed in HIV negative men based on differences in the clinical cohorts, laboratory methods, and specific cell subsets being analyzed in each of these studies. As has been reported previously for blood^22^, we recovered very few Tc17 cells from blood or rectal tissues in HIV positive men to enable analyses of this subset. The tissue-specific distribution of Th1, Th17, and Tc1 cells in HIV positive men was overall similar to HIV negative men (Fig. 2a,b).

**Figure 3:**
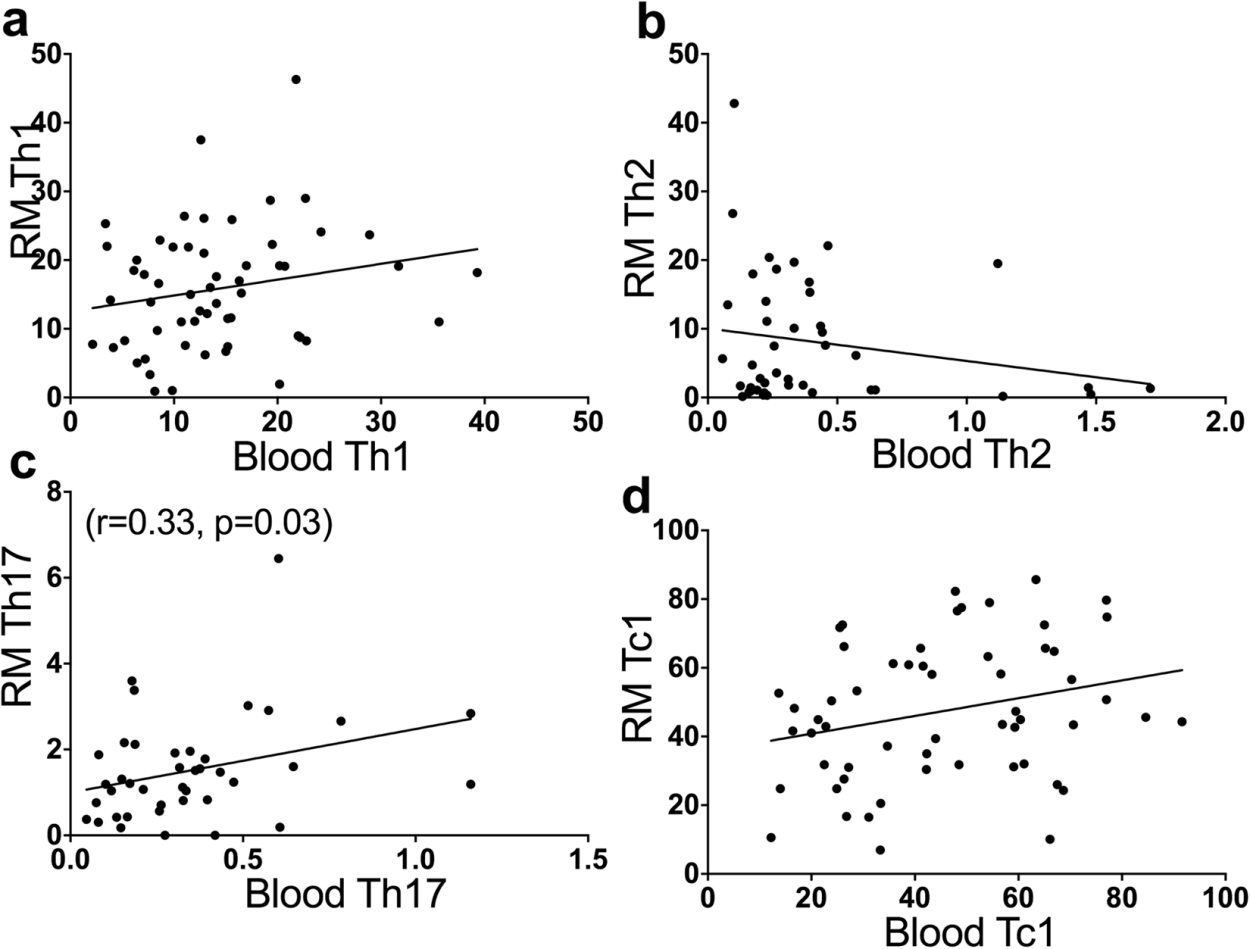
Correlation analysis of various Th and Tc subsets between peripheral blood and rectal mucosal (RM) compartments among HIV negative men. No significant correlation was seen for the Th1 (**a**), Th2 (**b**), or Tc1 (**d**) subsets. The Th17 subset (**c**) demonstrated a weakly positive correlation (r = 0.33, p = 0.03). Reduced frequencies of Tc2 and Tc17 cells in blood precluded the performance of correlation analyses of these cell subsets between tissue compartments. r = Spearman rank correlation coefficient; statistically significant findings were considered when p < 0.05.

Utilizing Spearman correlation analysis to detect similarities in the distribution of CD4 and CD8 T cell subsets between the peripheral blood and rectal mucosa in HIV negative men, none of the CD4 or CD8 T cell subsets showed significant correlation between the two compartments, with the exception of a weak correlation of the Th17 subset (Fig. 3). Likewise, there were no significant correlations between tissue compartmental Th1, Th17, or Tc1 cell subsets among the HIV positive cohort (data not shown). Thus, these findings suggest that rectal mucosa comprises an immune cell composition that is distinct from the peripheral blood.

### In the rectal mucosa of HIV negative men, Tc17 cells produce multiple pro-inflammatory cytokines in contrast to Th17 cells which demonstrate a more limited functional profile

**Figure 4:**
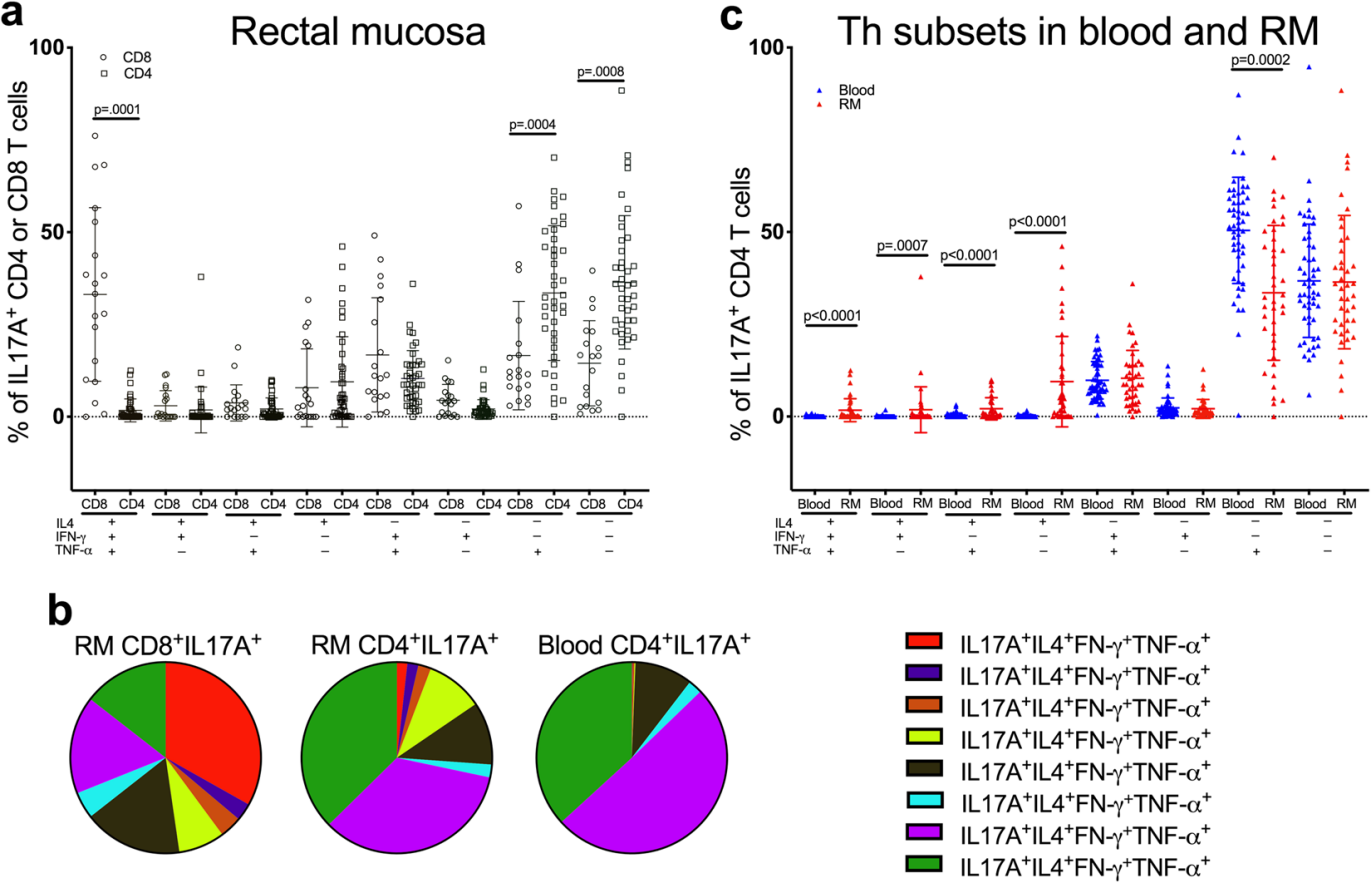
Polyfunctionality of IL-17-producing CD4 and CD8 T cells among HIV negative men. (**a**) Cytokine expression profiles based on IL-4, TNF-α, and IFN-γ secretion by IL-17A-producing CD4 and CD8 T cells in the rectal mucosa. (**b**) Pie graphs representing data from (**a**) showing that the majority of rectal mucosal IL-17A-producing CD8 T cells are multi-cytokine-producers compared to IL-17A-producing CD4 T cells which are more likely to be single IL-17A- or dual IL-17A^+^TNF-α^+^-producers. (**c**) Comparison of the frequencies of polyfunctional Th17 cells between blood (blue triangles) and RM (red triangles). Error bars represent means ± SD; statistically significant findings were considered when p < 0.05 (Wilcoxon matched-pairs signed rank test). A generalized Hotelling’s test^33^ (R package “GHT”) was used to compare the overall distribution of cytokine production cells between the Th and Tc subsets in RM (a, p = 0.002) and to compare differential cytokine production by the Th17 subsets between blood and RM compartments (c, p = 0.0001).

To better characterize the functional phenotypes of Tc17 and Th17 cells, we measured their ability to co-produce IFN-γ, TNF-α, and IL-4 using Boolean combination gating. For these analyses, we considered only those samples that contained a minimum of 100 IL-17A^+^ events. The frequency of blood Tc17 cell was low prompting exclusion from this analysis. The overall composition of cytokine production varied significantly between Tc17 and Th17 subsets in the rectal mucosa (p = 0.002). The majority of mucosal Tc17 cells produced multiple pro-inflammatory cytokines, with the largest proportion (33%) made up of quadruple cytokine-producers primed to secrete IL-17A, IFN-γ, TNF-α and IL-4 following activation (Fig. 4a,b). In addition, a significant proportion of the remaining Tc17 cells constituted IL-17A^+^IFN-γ^+^TNF-α^+^ triple-producers and IL-17A^+^TNF-α^+^ dual-producers. In contrast, despite a pattern of greater polyfunctionality of Th17 cells observed in the rectal mucosa compared to the blood, the majority of Th17 cells in both compartments were noted to be single IL-17A-producers or dual cytokine-producers (IL-17A^+^TNF-α^+^) (Fig. 4c; p = 0.0001 for the overall difference in distribution of cytokine production between blood and rectal mucosal Th17 cells). Consequently, these findings suggest that Tc17 cells are more likely to produce multiple pro-inflammatory cytokines compared to Th17 cells in the rectal mucosa. Yet, in comparing Th17 cell populations within the blood and rectal mucosal compartments, the mucosal cells have a greater overall likelihood of being multi-cytokine producers.

Similar to the results in HIV negative men, the majority of Th17 cells were single IL-17A cytokine-producers in the blood and rectal mucosal compartments (Fig. 2c,d) of HIV positive men. Nonetheless, Th17 cells in the rectal mucosa were more likely to be triple cytokine-producing (IL-17A, IFN-γ, and TNF-α) compared to the blood (p = 0.0049).

### Higher frequencies of pro-inflammatory Th17 and Tc17 cell subsets in the rectal mucosa correlate with an increase in mucosal HIV target cell availability in HIV negative men

**Figure 5:**
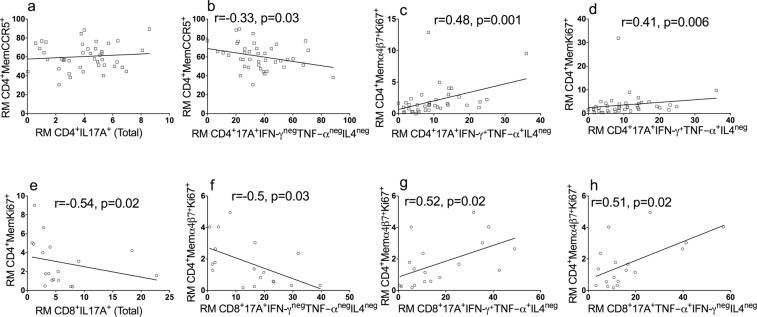
Association of proinflammatory cytokine-producing Th17 and Tc17 cells with HIV target cell population in the rectal mucosa (RM) in HIV negative men. (**a**) No correlation was seen with the total IL17A- producers. There was negative correlation observed between the frequency of CD4 memory CCR5^+^ cells and IL17A^+^ single cytokine-producing Th17 (**b**) cells. The IL17A^+^IFN-γ^+^TNF-α^+^IL4^neg^ cells showed a positive correlation with CD4 memory α4β7^+^Ki67^+^ (**b**) and CD4 memory Ki67^+^ cells (**d**). Unlike the total Th17 population, total Tc17 cells showed negative correlation with the proliferating memory CD4 T cells (**e**); the single IL17A-producing Tc17 showed negative correlation with proliferating and gut homing (α4β7^+^Ki67^+^) CD4 memory cells (**f**); the frequencies of memory CD4 T cells expressing α4β7^+^Ki67^+^ showed positive correlation with CD8^+^IL17A^+^IFN-γ^+^TNF-α^+^IL4^neg^ (**g**) and CD8^+^IL17A^+^TNF-α^+^IFN-γ^neg^IL4^neg^ (**h**), respectively. r = Spearman rank correlation coefficient; statistically significant findings were considered when p < 0.05.

To understand associations between polyfunctional Th17 and Tc17 subsets and the phenotype of memory CD4 T (CD4+ CD45RO+) cells in the rectum, which comprise the majority of CD4+ cells in the rectal mucosa, we measured the expression of CD38^+^ (indicator of activation status), Ki67^+^ (indicator of proliferation status), CCR5^+^ (co-receptor for HIV) and α4β7^+^Ki67^+^ (gut homing potential and ability to facilitate HIV infection) on unstimulated rectal mucosal memory CD4 T cells from HIV negative study participants. These markers were chosen to characterize the frequency of potential HIV target cells in the rectal mucosa. Notably, there was no significant association between total IL-17A-producing CD4 T cells and the frequency of HIV-susceptible memory CD4 T cells (CD4^+^CD38^+^, CD4^+^CCR5^+^, CD4^+^Ki67^+^ and CD4^+^α4β7^+^Ki67^+^ cells) in the rectal mucosa (Fig. 5a and Table S1). However, further analysis of Th17 subsets did reveal patterns of association with mucosal HIV target cell availability. The frequency of CD4^+^IL17A^+^ single cytokine-producing cells negatively correlated with the percentage of memory CD4^+^CCR5^+^ cells (r = −0.33; p = 0.03; Fig. 5b), while the percentage of CD4^+^IL17A^+^ cells that co-produced TNF-α^+^ and IFN-γ^+^ positively correlated with the percentage of memory CD4^+^α4β7^+^Ki67^+^ (r = 0.48, p = 0.001; Fig. 5c) and CD4^+^Ki67^+^ cells (r = 0.41, p = 0.006; Fig. 5d).

A similar analysis was conducted to evaluate for potential associations between rectal mucosal IL-17A-producing CD8 T cells (Tc17) and the proportion of available HIV target cells in HIV negative men (Fig. 5e–h). An increased frequency of the total Tc17 (CD8^+^IL17A^+^) cells was associated with fewer proliferating memory CD4 T cells (CD4^+^Ki67^+^) (r = −0.54, p = 0.02, Fig. 5e). There was also a negative correlation observed between single IL-17-producing CD8 T cells and α4β7^+^Ki67^+^ memory CD4 T cells (r = −0.5, p = 0.03, Fig. 5f). In contrast, increased frequencies of two polyfunctional Tc17 subgroups, IL-17A^+^IFN-γ^+^TNF-α^+^ and IL-17A^+^ TNF-α^+^ were associated with a higher proportion of mucosal α4β7^+^Ki67^+^ memory CD4 T cells (r = 0.52, p = 0.02 and r = 0.51, p = 0.02, respectively; Fig. 5g,h). For the HIV positive cohort, a corresponding analysis demonstrated no correlation between the frequencies of any of the mucosal Th17 subsets (CD4^+^IL-17A^+^IFN-γ^+^TNF-α^+^, CD4^+^IL-17A^+^IFN-γ^+^ TNF-α^neg^, CD4^+^IL-17A^+^TNF-α^+^ IFN-γ^neg^, or CD4^+^IL-17A^+^IFN-γ^neg^TNF-α^neg^) and the proportion of memory CD4^+^CCR5^+^ and CD4^+^Ki67^+^ cell subtypes in the rectal mucosa (data not shown).

In summary, the findings in HIV negative men suggest that increased frequencies of polyfunctional, pro-inflammatory Th17 and Tc17 cells in the rectal mucosal immune environment may promote phenotypic expression of CD4 T cells that enhance HIV susceptibility, whereas single IL-17A-producing CD4 and CD8 T cells appear to be associated with decreased frequency of HIV target cells in the rectal mucosa.

## Discussion

In this study, we describe an immune cell composition of the rectal mucosa in HIV negative individuals that is distinct from the peripheral blood compartment in that it is enriched with IL-17-producing CD4 and CD8 T cells. In comparing the functional profiles of Tc17 and Th17 cells in the rectal mucosa, we found that the Tc17 cell subset is more likely to be polyfunctional with the majority of Tc17 cells being quadruple- and triple-cytokine-producers. In contrast to the Th17 cell population in the peripheral blood compartment, the rectal mucosal Th17 cell subset exhibits higher rates of polyfunctionality with dual-cytokine-producing CD4^+^IL-17A^+^TNF-α^+^ cells being the most common multi-cytokine-producing cell type. Additionally, among the HIV negative cohort, we demonstrated that higher frequencies of pro-inflammatory, multi-cytokine-producing Th17 and Tc17 cell subsets were associated with increased rectal mucosal HIV target cell availability, while single IL-17A-producing Th and Tc cell subsets were associated with reduced frequencies of mucosal HIV target cells in the rectum.

To our knowledge, this study is among the few reports^23^ to compare the distribution of multiple Th and Tc cell subsets between the blood and rectal mucosal compartments in human subjects. Similar to the Th17 cell subset, Tc17 cells were shown to predominantly reside in the rectal mucosa in HIV negative men, suggesting that they also play an important role in intestinal mucosal barrier protection. We hypothesize that this finding is likely related to co-regulation as both subsets differentiate into IL-17 producers following exposure to IL-6 and TGF-β^24^. Th2 cells were also more heavily distributed in the rectal mucosa, which presumably corresponds with their primary role in defense against parasitic pathogens. Notably, except for a weak correlation of the Th17 subset, none of the other Th or Tc subsets (Th1, Th2, Tc1, Tc2, or Tc17) showed any significant correlation between the two compartments. These findings display important differences in the immune cell composition of the peripheral blood and rectal mucosal tissue compartments, thus illustrating the inherent limitations that exist when immunologic characteristics of the peripheral blood are considered representative of the rectal mucosal immune environment.

In this study, we also observed that specific effector functions exhibited by T cells vary depending on their tissue localization. Compared to Th17 cells isolated from the blood compartment, the rectal mucosal population was more likely to produce multiple pro-inflammatory cytokines. Our data from HIV negative subjects demonstrated that approximately 64% of the total Th17 cells in the rectal mucosa were multi-cytokine-producing with the largest proportion being dual-cytokine-producing CD4^+^IL-17A^+^TNF-α^+^ cells. This finding likely reflects higher levels of antigen exposure at sites of tissue residence where these cells carry out their mucosal barrier functions. These multi-cytokine-producing Th17 cells are considered pathogenic in certain contexts as they have been implicated in the development of autoimmune disease through the secretion of pro-inflammatory cytokines, including TNF-α, IFN-γ, and granulocyte-macrophage colony-stimulating factor (GM-CSF)^11,25^. During HIV transmission, both single-cytokine-producing Th17 cells and multi-cytokine-producing Th17/Th1 cells are permissive to HIV infection^11,12,17^. However, there may be a substantial difference in the extent to which these cell subsets contribute to HIV transmission. In previous studies evaluating HIV susceptibility among Th17 subsets, Th17/Th1 cells exhibited greater expression of CCR5 and the HIV binding molecule, α4β7, compared to single-cytokine-producing Th17 cells, and this increased expression was associated with greater susceptibility to infection with R5-tropic virus^12,17^. Our data support these findings by showing a strong positive correlation between certain proinflammatory cytokine-producing Th17 subsets and activated CD4 T cells in the rectal mucosa. The mechanisms prompting differentiation into pathogenic vs non-pathogenic Th17 cells in mucosal tissues remain elusive. Further characterization of these pathogenic Th17 cells, especially of their origin and effector functions, is needed and may ultimately provide insight into their role in HIV transmission and disease progression.

Increased frequencies of Tc17 cells have also been reported in patients with active systemic lupus erythematosus and psoriasis, supporting their role in the pathogenesis of these diseases^24,26^. Our data show that the majority of Tc17 cells isolated from the rectal mucosa of HIV negative men are polyfunctional with the largest proportion being quadruple-cytokine-producers. The capacity to produce multiple cytokines apart from IL-17A, was observed by Hamada *et al*.^21^ who noted that protection afforded by Tc17 cells during lethal influenza challenge experiments in mice occurred through the recruitment of neutrophils into the lung and not from direct cytolysis. The specific role of these cells is not yet clearly defined in the rectal mucosa, although what is known about their functional activity closely parallels the effector functions of Th17 cells and differs significantly from the cytotoxic mechanisms of Tc1 and Tc2 subsets.

The release of pro-inflammatory cytokines by IL-17-producing Th and Tc cells appears to promote the infiltration of neutrophils and other innate immune cells into mucosal tissues^27^. Although causality cannot be established, we found that higher frequencies of pro-inflammatory Th17 and Tc17 cells were associated with greater mucosal HIV target cell availability in HIV negative men. It is possible that the release of pro-inflammatory mediators by IL-17-producing cells either directly promote the influx of highly HIV-susceptible CD4 T cells into the rectal mucosa or potentially these target cells are summoned indirectly through chemoattractant signaling by neutrophils and other innate immune cells. Alternatively, considering that Th17 cells are in and of themselves HIV target cells^28^, these findings may reflect that certain subsets of polyfunctional Th17 cells are more likely to be in an activated state with increased expression of HIV susceptibility markers. Either through their role in potentiating mucosal inflammation or possibly due to the expression of phenotypic features that correlate with HIV susceptibility, our results suggest that pro-inflammatory Th17 and Tc17 cells likely enhance HIV transmission.

While not directly comparable due to differences in study cohorts, our findings from the HIV positive men closely corresponded with those from the HIV negative group in terms of CD4 and CD8 T cell distributions between the peripheral blood and rectal mucosal compartments. Additionally, in both groups there were higher proportions of single IL-17A-producing and dual-cytokine-producing (CD4^+^IL-17A^+^TNF-α^+^) CD4 T cells within both tissue compartments compared with lower frequencies of other multi-cytokine-producing Th17 subsets. Yet, compared to the findings among the HIV negative cohort, no correlation was observed between multi-cytokine-producing Th17 cells and highly HIV-susceptible T cells in the rectal mucosa of a smaller sample of HIV positive subjects. As the majority of HIV positive subjects in our study were chronically-infected with HIV and had achieved immune reconstitution and virologic suppression, they would be expected to have lower levels of immune activation and CD4 proliferation^29^, which may explain these findings. In addition, there are likely distinct differences in the rectal mucosal immune environments of HIV positive compared to HIV negative individuals that extend beyond the T cell compartment, and the data presented here are hypothesis-generating and will be valuable in the design of future studies that examine the relationship between IL-17-producing cells and mucosal T cell activation.

Interestingly, we found reduced frequencies of blood and rectal mucosal Tc17 cells isolated from HIV positive subjects, a finding consistent with results from a prior study which demonstrated lower levels of circulating Tc17 cells within the peripheral blood compartment of HIV positive individuals regardless of treatment status or level of immune reconstitution compared to healthy controls^22^. As there is limited knowledge regarding the potential role of rectal mucosal Tc17 cells during HIV transmission and the ways in which this population may be impacted by HIV disease progression, further study is warranted to more comprehensively evaluate the functional characteristics of this subset.

The results of this study are mainly descriptive, and the cross-sectional study design limits our ability to establish a causal relationship between polyfunctional IL-17-producing T lymphocytes and HIV target cell availability in the rectal mucosa. As the blood and rectal mucosal specimens have been collected for use in studies that evaluate the effects of receptive anal intercourse by comparing the rectal mucosal immune environment between men who have sex with men (MSM) and male controls^30^, only male study participants were included and further investigation will be needed to determine whether differences in sex may influence these findings. Additionally, this study was not powered to examine the effects of specific sexual behaviors on functional T cell subsets in the mucosal tissues due to our strict inclusion criteria of 100-cytokine positive cells. Therefore, we cannot exclude the possibility of finding variable outcomes in regard to the distribution and functional phenotypes of Th17 and Tc17 cells based on sex or behavioral factors. Future research is warranted to more comprehensively evaluate environmental, pharmacological, and behavioral factors, including co-infection with rectal STIs, antimicrobial use, substance abuse, etc. that may influence GI mucosal inflammation and immune cell composition.

In summary, we have defined the distribution of Th and Tc cells subsets within the rectal mucosa and have demonstrated an association between increased frequencies of multi-cytokine-producing Th17 and Tc17 cells and HIV target cell availability in the rectal mucosa of HIV negative men. Currently, it is not known to what extent, if any, the pro-inflammatory effects of these polyfunctional cells may influence HIV transmission and pathogenesis. Therefore, further studies will be required to explore the mechanisms that regulate inflammation within the rectal mucosa. Gaining insight into the immune correlates of HIV transmission at mucosal sites may contribute to the development and optimization of biomedical HIV prevention interventions, including an effective HIV vaccine.

## Methods

### The clinical cohorts

In this manuscript, we conducted a sub-analysis of a parent study^31^ that enrolled 62 HIV negative men to compare the rectal mucosal immune environment between men who have sex with men who were engaging in condomless receptive anal intercourse and men who had never engaged in anal intercourse. The Institutional Review Board (IRB) at Emory University approved this study and all experiments were performed in accordance with relevant guidelines and regulations. An informed consent was obtained from all participants, according to the IRB guidelines. Participants were healthy men, aged 18–45 years from the Atlanta community. Men who were determined by the principal investigator to be high risk for rectal biopsy procedures due to medical comorbidities or who intended to take pre-exposure prophylaxis during the study were not enrolled. The parent study collected blood and rectal biopsy specimens from two study visits separated by a median of 9 weeks. All biopsies were collected ~3–10 cm from the anal verge via rigid sigmoidoscopy with no prior bowel preparation. We also analyzed data from a separate study protocol that enrolled HIV positive men who have sex with men (n = 21) 18–24 years of age for blood and rectal biopsies from a single study visit with similar study procedures as outlined above.

### Blood and rectal mucosal mononuclear cell phenotyping

For the HIV negative cohort, blood collected in sodium citrate tubes was processed by Ficoll density gradient to separate peripheral blood mononuclear cells, and 5 pinch biopsies from the rectal mucosa were processed by collagenase digestion to separate mucosal mononuclear cells as described previously^32^. Mononuclear cells thus isolated from the blood and rectal biopsies were stained with live/dead marker and antibodies specific to CD3, CD4, CD8, CCR7, CD45RO, CD45, CCR5, α4β7, CD38, and HLA-DR as described previously^31^. Biopsy specimen processing was similar for the HIV positive cohort except the CCR7 and CD38 antibodies were not included in the panel for this group.

### Intracellular cytokine staining

One million cells were stimulated with 25 ng/ml of PMA and 500 ng/ml of Ionomycin in the presence of BrefeldinA (5 μg/ml; Sigma-Aldrich; St. Louis,MO) and Golgi stop (0.5 μl/ml; BD Pharmingen; San Jose, CA) and incubated for 4 h at 37 °C in the presence of 5% CO_2_. At the end of stimulation, cells were stained with live/dead marker and surface markers, anti-CD3, anti-CD4, and anti-CD8. Following fixation and permeabilization cells were stained for intracellular cytokines with IL-17A, IFN-γ, TNF-α, and IL4 antibodies as described previously^31^. For the HIV positive cohort, non-stimulated and stimulated blood and rectal mucosal mononuclear cells were stained for intracellular cytokine production with IL-17A, IFN-γ, and TNF-α antibodies; IL-4 was not included in the ICS panel based on the study protocol.

### Data analyses

The flow data was analyzed on FlowJo10 software (Treestar Ic. CA). In defining the Th or Tc subsets, 100 cytokine-specific positive events was used as the minimum number of events for inclusion in the study in order to ensure rigorous results, and Boolean analysis was used to categorize polyfunctional subsets. Polyfunctionality of IL-17-producing CD4 and CD8 T cells in the HIV negative cohort was defined by depicting each possible combination of IFN-γ, TNF-α and IL-4 by IL-17A^+^ cells with Boolean analysis. Likewise, for the HIV positive cohort, mutli-cytokine-producers were defined based on IFN-γ and TNF-α expression by IL-17A^+^ cells using Boolean analysis. Tissue-specific prevalences of T cell subsets were analyzed using the Wilcoxon matched-pairs signed rank test and correlations between the cell subsets were assessed by non-parametric Spearman correlation. For testing the (multivariate) compositional data for paired samples, we adopted the Generalized Hotelling’s test^33^ (R package “GHT”). Data plots and statistical analyses were performed using Prism 7 (GraphPad Software, La Jolla, CA).

## Supplementary information

Supplementary Files
