# Author Correction: Distribution of Functional CD4 and CD8 T cell Subsets in Blood and Rectal Mucosal Tissues

**DOI:** 10.1038/s41598-020-67062-x

**Published:** 2020-06-17

**Authors:** Praveen Kumar Amancha, Cassie G. Ackerley, Chandni Duphare, Mark Lee, Yi-Juan Hu, Rama R. Amara, Colleen F. Kelley

**Affiliations:** 1 The Hope Clinic of the Emory Vaccine Research Center, Division of Infectious Diseases, Department of Medicine, Emory University School of Medicine, Decatur, GA 30030 United States; 2 Present Address: Pfizer Pharmaceuticals, Cambridge, MA United States; 3 Yerkes National Primate Research Center, Emory Vaccine Center, Atlanta, GA 30329 United States; 4 Department of Biostatistics and Bioinformatics, Rollins School of Public Health, Emory University, Atlanta, GA 30322 United States

**Correction to**: *Scientific Reports* 10.1038/s41598-019-43311-6, published online 06 May 2019

The Article contains errors in Figure 4, where the figure keys are incorrect. The correct Figure 4 appears below as Figure 1.

**Figure 1:**
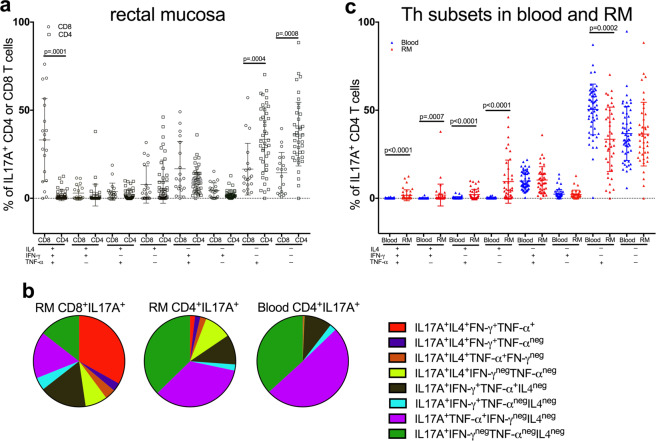
.

