# Supplementary material for: Distribution of Functional CD4 and CD8 T cell Subsets in Blood and Rectal Mucosal Tissues
Source: Sci Rep. 2019 May 6;9:6951. doi: 10.1038/s41598-019-43311-6 (PMC6502862; doi:10.1038/s41598-019-43311-6)
Supplement: Supplementary file 1 — Supplementary Files [file 41598_2019_43311_MOESM1_ESM.pdf]

Distribution of Functional CD4 and CD8 T cell Subsets in Blood and Rectal Mucosal Tissues

Praveen Kumar Amancha*[1], Cassie G. Ackerley*[1], Chandni Duphare[2], Mark Lee[2], Yi-Juan Hu[3], Rama R. Amara[2] and Colleen F. Kelley[1]

Figure S1: Representative FACS plots for intracellular cytokine staining assays. The no stimulation control (NS) and PMA/Ionomycin stimulated profile is shown here on CD4 (left) and on CD8 (right).

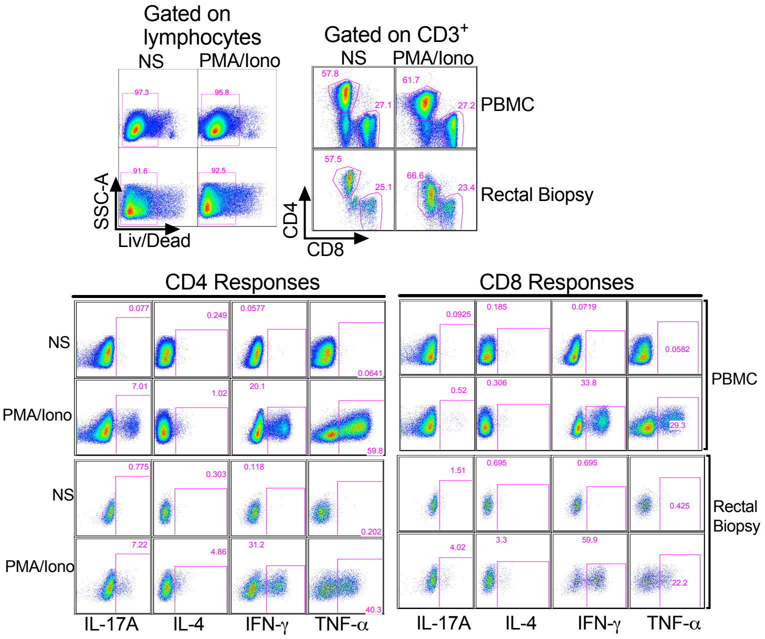

Table S1: Correlation analysis of polyfunctional Th17 and Tc17 populations and HIV target cell populations in the RM of HIV negative men

	Memory $CD4^{+}CCR5^{+}$	Memory $CD4^{+}Ki67^{+}$	Memory $CD4^{+}CD38^{+}$	Memory $CD4^{+}\alpha4\beta7^{+}Ki67$
Total $CD4^{+}IL17A^{+}$	ns	ns	ns	ns
$CD4^{+}IL17A^{+}IFN\text{-}\gamma^{neg}TNF\alpha^{neg}IL4^{neg}$	* (r= -0.33, p=0.03)	ns	ns	ns
$CD4^{+}IL17A^{+}IFN\text{-}\gamma^{+}TNF\alpha^{neg}IL4^{neg}$	ns	ns	ns	ns
$CD4^{+}IL17A^{+}TNF\text{-}\alpha^{+}IFN\text{-}\gamma^{neg}IL4^{neg}$	ns	ns	ns	ns
$CD4^{+}IL17A^{+}IFNg\text{-}\gamma^{+}TNF\text{-}\alpha^{+}IL4^{neg}$	ns	** (r= 0.41, p=0.006)	ns	** (r= 0.48, p=0.001)
$CD4^{+}IL17A^{+}IL4^{+}IFN\text{-}\gamma^{neg}TNF\alpha^{neg}$	ns	ns	ns	ns
$CD4^{+}IL17A^{+}IL4^{+}IFNg\text{-}\gamma^{+}TNF\alpha^{neg}$	ns	ns	ns	ns
$CD4^{+}IL17A^{+}IL4^{+}TNF\text{-}\alpha^{+}IFN\text{-}\gamma^{neg}$	ns	ns	ns	ns
$CD4^{+}IL17A^{+}IL4^{+}IFNg\text{-}\gamma^{+}TNF\text{-}\alpha^{+}$	ns	ns	ns	ns
	RM_CD4MemCCR5	RM_CD4 MemKi67	RM_CD4 MemCD38	RM_CD4 Mema4b7_Ki67
Total $CD8^{+}IL17A^{+}$	ns	* (r= -0.54, p=0.02)	ns	ns
$CD8^{+}IL17A^{+}IFN\text{-}\gamma^{neg}TNF\alpha^{neg}IL4^{neg}$	ns	ns	ns	* (r= -0.50, p=0.03)
$CD8^{+}IL17A^{+}IFN\text{-}\gamma^{+}TNF\alpha^{neg}IL4^{neg}$	ns	ns	ns	ns
$CD8^{+}IL17A^{+}TNF\text{-}\alpha^{+}IFN\text{-}\gamma^{neg}IL4^{neg}$	ns	ns	ns	* (r= 0.51, p=0.02)
$CD8^{+}IL17A^{+}IFNg\text{-}\gamma^{+}TNF\text{-}\alpha^{+}IL4^{neg}$	ns	ns	ns	* (r= 0.52, p=0.02)
$CD8^{+}IL17A^{+}IL4^{+}IFN\text{-}\gamma^{neg}TNF\alpha^{neg}$	ns	ns	ns	* (r= -0.57, p=0.01)
$CD8^{+}IL17A^{+}IL4^{+}IFNg\text{-}\gamma^{+}TNF\alpha^{neg}$	ns	ns	ns	* (r= -0.514, p=0.03)
$CD8^{+}IL17A^{+}IL4^{+}TNF\text{-}\alpha^{+}IFN\text{-}\gamma^{neg}$	ns	ns	ns	ns
$CD8^{+}IL17A^{+}IL4^{+}IFNg\text{-}\gamma^{+}TNF\text{-}\alpha^{+}$	ns	ns	ns	ns
